# Systematic review of methods used in prediction models with recurrent event data

**DOI:** 10.1186/s41512-024-00173-5

**Published:** 2024-08-06

**Authors:** Victoria Watson, Catrin Tudur Smith, Laura J. Bonnett

**Affiliations:** Department of Health Data Sciences, University of Liverpool, Liverpool, UK

## Abstract

**Background:**

Patients who suffer from chronic conditions or diseases are susceptible to experiencing repeated events of the same type (e.g. seizures), termed ‘recurrent events’. Prediction models can be used to predict the risk of recurrence so that intervention or management can be tailored accordingly, but statistical methodology can vary. The objective of this systematic review was to identify and describe statistical approaches that have been applied for the development and validation of multivariable prediction models with recurrent event data. A secondary objective was to informally assess the characteristics and quality of analysis approaches used in the development and validation of prediction models of recurrent event data.

**Methods:**

Searches were run in MEDLINE using a search strategy in 2019 which included index terms and phrases related to recurrent events and prediction models. For studies to be included in the review they must have developed or validated a multivariable clinical prediction model for recurrent event outcome data, specifically modelling the recurrent events and the timing between them.

The statistical analysis methods used to analyse the recurrent event data in the clinical prediction model were extracted to answer the primary aim of the systematic review. In addition, items such as the event rate as well as any discrimination and calibration statistics that were used to assess the model performance were extracted for the secondary aim of the review.

**Results:**

A total of 855 publications were identified using the developed search strategy and 301 of these are included in our systematic review. The Andersen-Gill method was identified as the most commonly applied method in the analysis of recurrent events, which was used in 152 (50.5%) studies. This was closely followed by frailty models which were used in 116 (38.5%) included studies. Of the 301 included studies, only 75 (24.9%) internally validated their model(s) and three (1.0%) validated their model(s) in an external dataset.

**Conclusions:**

This review identified a variety of methods which are used in practice when developing or validating prediction models for recurrent events. The variability of the approaches identified is cause for concern as it indicates possible immaturity in the field and highlights the need for more methodological research to bring greater consistency in approach of recurrent event analysis. Further work is required to ensure publications report all required information and use robust statistical methods for model development and validation.

**PROSPERO registration:**

CRD42019116031.

## Introduction

A chronic condition is as a long-term medical condition, such as epilepsy and asthma. Patients with such conditions are at risk of multiple recurrences over their lifetime and often these chronic conditions or diseases have no cure [1, 2]. Despite this, there may be medications and/or therapies available which can help control the chronic condition. These can improve patients’ quality of life and independence by improving their ability to perform day-to-day activities such as social activities, exercising and work. Chronic diseases contribute to the largest proportion of diseases and this is expected to rise with the aging population [3]. The World Health Organization (WHO) reported diabetes mellitus, cardiovascular and chronic respiratory diseases, cancer and stroke as the ‘big 5’ chronic diseases worldwide [4].

It can be challenging for clinicians and patients to make decisions regarding starting and stopping treatments for chronic conditions as outcomes are often heterogeneous and it is necessary to balance the benefits and harms of treatments. Clinical prediction models can help inform treatment choice and guide patient counselling [5]. They combine multiple pieces of patient information to predict a clinical outcome for people with a particular medical condition [6].

Many prediction models for recurrent conditions estimate which patient subgroups have higher recurrence risks, based only on limited information about prior events, such as time from diagnosis to first event. Although such models can be useful, they do not fully utilize the information that can be collected from patients on the history of all previous events, and they cannot be updated whenever a patient has a recurrence [7]. Therefore, it is necessary to consider methods for modelling all events along a patient’s journey to better predict outcome and therefore better inform discussions between patients and clinicians regarding treatment strategies.

## Aims

The aims of this systematic review were to (i) identify and describe existing methodology being applied for the development and validation of prediction models for recurrent event outcome data, (ii) to informally assess the quality of analysis reported, including the use of model performance measures, in the development and validation of prediction models for recurrent event data.

## Methods

Full methodological details are available in the associated protocol [1].

### Search strategy

A search strategy was developed to ensure identification of as many studies as possible relevant to the systematic review; the Ingui search filter [8] for prediction models was combined with terms associated with statistical models for recurrent events, as recommended by a specialist librarian. The database used to identify studies was the Medical Literature Analysis and Retrieval System Online (MEDLINE). The search strategy is described in Table 11 in Appendix 1, and was run on 24th October 2019.

### Selection criteria

Studies chosen for inclusion in the review were carefully assessed against pre-defined inclusion criteria. The systematic review focussed on methodology used to develop and validate multivariable prediction models for recurrent events as a result of a chronic condition or disease. A recurrent event was defined as an event of the same type occurring multiple times for the same individual. For example, repeated seizures in people with epilepsy, repeated hospitalisations for people with heart conditions or recurrent urinary tract infections. Papers which applied recurrent event analysis methods to areas which were not applicable to a chronic condition or disease were not included. Examples of these include papers which analysed juvenile data for repeat offenders or motorcycle/car crashes.

For studies to be included in the review, they must have developed or validated a multivariable prediction model for recurrent event data predicting the risk of future recurrences. Included studies had to include both the number of recurrent events and also the timing between them as part of the model. Studies which only analysed the time to the first event only using a standard Cox model for example, or studies which analysed only the number of events using a Poisson or Negative Binomial model for example were not included. Similarly, studies considering only one prognostic factor were excluded.

### Study design

No restrictions were placed on the data collection approach used in studies, for example both retrospective and prospective studies were included.

### Setting and study population

No restriction was placed on the setting the study was conducted in nor did the search strategy focus on a certain study population regarding age group or ethnicity.

### Study selection

The study selection process consisted of two independent reviewers, who first screened titles and abstracts using pre-defined screening criteria.

Full texts were then obtained and were screened by the two independent reviewers separately against full eligibility criteria. Relevant texts were translated where deemed necessary when considering non-English texts.

Assessments between reviewers were discussed, and any discrepancies resolved. Reviewers’ decisions and reasons for exclusion were recorded.

### Data extraction

A detailed data extraction form was developed and piloted on 10 studies before it was finalised for use throughout the systematic review. The data extraction form collected information about the statistical method used to analyse recurrent events. Characteristics such as the country the study was conducted in and the dates the study took place over were also collected, as was the medical condition under consideration, the design of the study (Randomised Controlled Trial (RCT), cohort or case–control for example), and length of follow-up. The number of patients, the number of recurrent events and the number of patients who experienced recurrent events were extracted if provided.

Information regarding discriminatory statistics which examine the models’ ability to distinguish between those who had the event and those who did not were assessed, for example C-statistics, was extracted for each study. Similarly, information about the models’ calibration performance, which assesses the agreement between the observed probability to the predicted probability of risk, was extracted where available [2, 3].

Studies were categorised according whether the model was internally validated and/or externally validated.

### Quality of analysis assessment

As the priority of the review was to describe statistical methodology, we did not complete a full quality assessment for each study. However, we did assess the ‘analysis’ domain from the ‘Prediction study Risk Of Bias Assessment Tool’ (PROBAST) [9] as an informal assessment of the quality of analysis. This included an assessment of how the prognostic factors to be included in the final model were chosen, and how prognostic factors were entered into the model. Missing data was also assessed, for example the overall completeness of the data and the numbers lost to follow up (LTF). Approaches for handling missing data (imputation or complete case analysis) were extracted. The source of the data was also recorded to assess potential bias, whether it be a cohort study, case–control or a RCT for example.

## Results

### Included studies

**Fig. 1:**
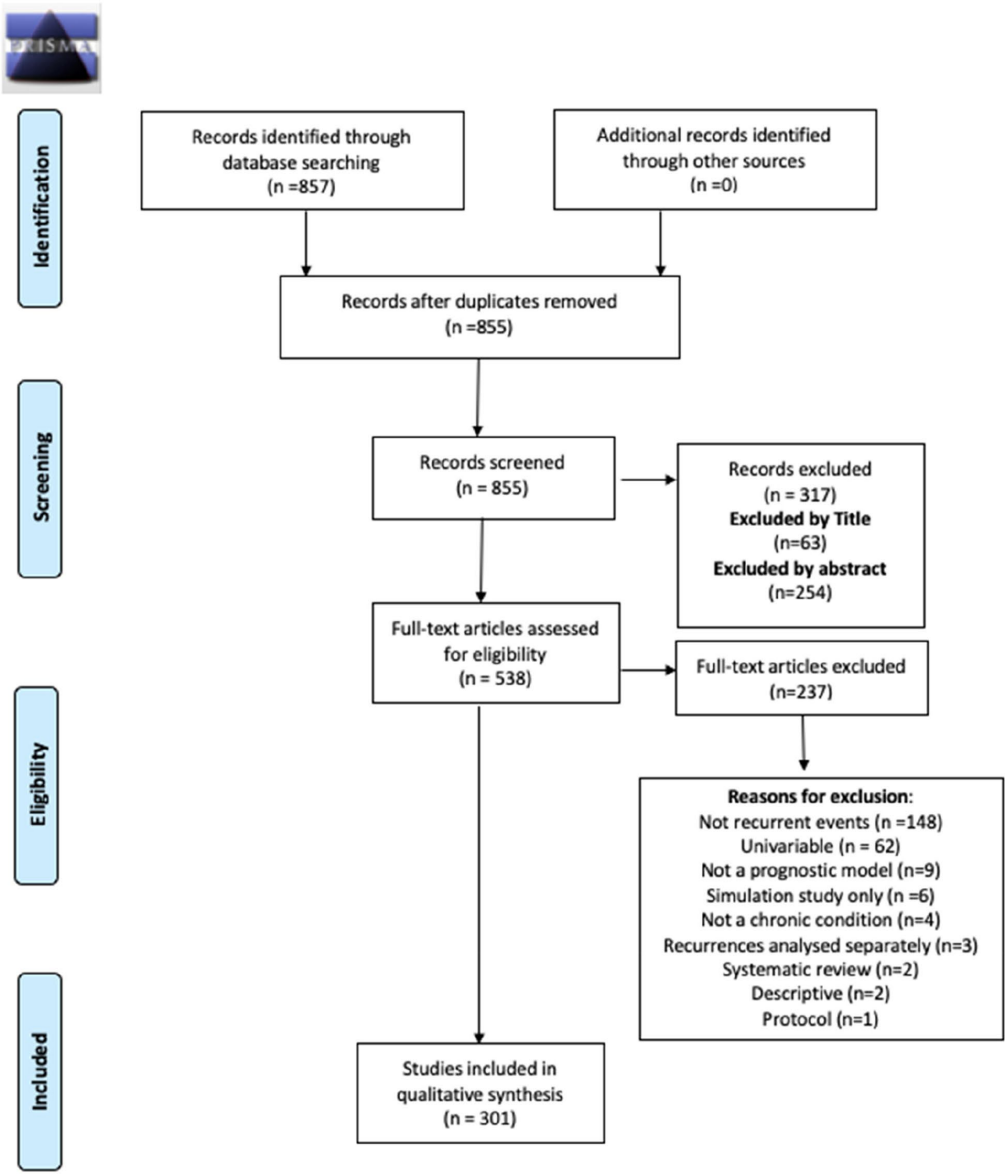
Completed PRISMA flow chart

By applying the search strategy, 855 papers were identified and screened (Fig. 1). Of these, 63 were excluded by title and 254 by abstract, leaving 538 to be assessed using the full-text. Of these, 237 papers were excluded after a full-text review leaving 301 papers to be included in the final review. A full list of included papers can be found in Table 12 in Appendix 2.

The 301 studies were published across a 34-year span, from 1985 to 2019. Cardiology was found to be the most frequently reported clinical area in 62 (20.6%) studies, for example studies which use these methods to analyse recurrent heart failure related admissions. Oncology studies were the second most applied area with 45 (15.0%) studies modelling tumour recurrences, such as recurrences of breast cancer [10–15], bladder cancer [16–19], rectal cancer [20–22] and oesophageal cancer [23] amongst other cancer types [24, 25]. The full list of clinical areas can be seen in Table 13 in Appendix 3.

The majority of studies, 173 (57.5%), used data from a cohort design. The remaining studies used data from RCTs (55 (18.3%)), case–control studies (12 (4.0%)) or cross-sectional studies (7 (2.3%)). Model development was the primary focus in 45 (15.0%) studies, rather than a primary objective of the paper to report analysis results of a clinical dataset.

A detailed summary of the included studies according to the aims of the review is detailed below.

### Statistical approaches to modelling recurrent events

The most frequently reported method for analysing recurrent events was the Andersen-Gill (AG) model [26], which was used in 152 (50.5%) of the 301 included papers (Table 1). This is an extension of the Cox model using robust standard errors to account for within subject heterogeneity between recurrence times within individuals. Frailty models [27] were used by 116 (38.5%) studies. Frailty models for recurrent event data also compromise of a Cox model analysing time to event data, but instead of using robust standard errors to account for within subject heterogeneity, random effects are added to the model. These random effects are referred to as the frailty variable in the model [27, 28]. A variety of frailty models were applied depending on distribution and these are summarised in Table 1. The most frequent was the gamma frailty model in 63 (20.9%) studies.

**Table 1:** Summary of methods identified from the data extraction

Method	Number (%) of included studies
Recurrent event methods	
Andersen-Gill (AG) [26]	152 (50.5%)
Frailty Model [27]:^a^	116 (38.5%)
Gamma	63 (20.9%)
Unspecified	35 (11.6%)
Gaussian	18 (6.0%)
Log-Normal	15 (5.0%)
Weibull	10 (3.3%)
Exponential	8 (2.7%)
Log-Logistic	3 (1.0%)
Poisson	3 (1.0%)
Compound Poisson	1 (0.3%)
Gompertz	1 (0.3%)
Logistic	1 (0.3%)
Prentice, Williams and Peterson Models [29]:^b^	41 (13.6%)
Prentice, Williams and Peterson-Total Time (PWP-TT)	27 (9.0%)
Prentice, Williams and Peterson-Gap Time (PWP-GT)	22 (7.3%)
Wei, Lei and Weissfeld (WLW) [30]	33 (11.0%)
Bayesian Methods	11 (3.7%)
Multi-State Model (MSM)	9 (3.0%)
Lin, Wei, Ying and Yang (LWYY) [31]	2 (0.7%)
Lee, Wei and Amato (LWA) [32]	1 (0.3%)
Lawless and Nadeau marginal model (LN) [33]	1 (0.3%)
Liang, Self and Chang (LSC) [34]	1 (0.3%)
Multilevel Survival Model [35]	1 (0.3%)
Papers which used multiple recurrent event methods	48 (15.9%)

^a^Some papers applied more than one type of frailty model

^b^Some papers applied both the PWP-TT and PWP-GT variation

There were 48 (15.9%) papers identified which used more than one method to analyse recurrent events.

### Quality of analysis assessment

Selected aspects of the PROBAST ‘analysis’ domain (domain 4), as described in the methods section, are now considered. The results for these can be found in Tables 2, 3, 4, 5, 6, 7, 8 and 9.

*Were there a reasonable number of participants with the outcome? (PROBAST 4.1)*

**Table 2:** PROBAST 4.1 results

**Category**	**Results**^a^
Total number of recurrent events reported	227 (75.4%)
Number of patients who experienced recurrent events reported	191 (63.5%)
Person years of follow-up reported directly	31 (10.3%)
Person years of follow-up approximated using the median length of follow-up	99 (32.9%)
Person years of follow-up approximated using the mean length of follow-up	42 (14.0%)
Event rate reported	114 (37.9%)
Event rate could be calculated manually using either person years or the mean/median length of follow-up	134 (44.5%)
Median (IQR) event rate	26.1 (5.9–59.3) per 100-person years^b^
EPV could be calculated	216 (71.8%)
Inadequate EPV of less than 20	27 (12.5%)^c^
Median (IQR) EPV	128.6 (33.5–419.5)^c^

^a^Results are number (%) of included studies unless stated otherwise

^b^Result is calculated from the 248 studies where the event rate was reported or could be calculated

^c^Result is calculated from the 216 studies where the EPV could be calculated

**Table 3:** PROBAST 4.2 results

Category	Number (%) of included studies
Categorisation of continuous predictors	62 (20.6%)

**Table 4:** PROBAST 4.3 results

Category	Number (%) of included studies
All Enrolled participants included in the analysis	229 (76.1%)
Where participants were excluded, the authors have justified the reasons for doing so	72 (23.9%)

**Table 5:** PROBAST 4.4 results

Method for handling missing data	Number (%) of included studies
Complete case analysis	46 (15.3%)
Multiple imputation	19 (6.3%)
Number of imputations reported	11 (57.9%)^a^
Last observation carried forward (LOCF)	5 (1.7%)

^a^Percentage calculated from the 19 studies where multiple imputation was used

**Table 6:** PROBAST 4.5 results

Category	Number (%) of included studies
Univariable screening	44 (14.6%)
Use of stepwise regression	23 (7.6%)

**Table 7:** PROBAST 4.7 results

Category	Number (%) of included studies
Internal validation:	
Calibration statistics	37 (12.3%)
Discrimination measures	30 (10.0%)
Several measures for calibration and discrimination reported	75 (24.9%)
External validation	3 (1.0%)

**Table 8:** PROBAST 4.8 results

Category	Number (%) of included studies
Model overfitting and optimism accounted for	74 (24.6%)
Bootstrap resampling used	20 (6.6%)
Cross validation methods used	8 (2.7%)

**Table 9:** PROBAST 4.9 results

Category	Number (%) of included studies
Number of predictors and levels for each reported	297 (98.7%)
Full results for all included predictors reported	202 (67.1%)

PROBAST states that the Events per Variable (EPV) included in a model should be greater than or equal to 20 for studies to have less chance of overfitting and thus be graded as low risk of bias [9]. The number of papers which report the EPV can be found in Table 2. If the EPV was not reported, it was calculated manually where possible by using the reported person years of follow-up. If the person years of follow-up was not reported, the EPV was approximated using either the mean or median length of follow-up. The number of events per 100-person years was calculated by dividing the number of recurrent events overall in the study by the total number of person years of follow-up and multiplied by 100. Where the EPV could not be calculated, it was not clear how many predictor levels had been included in the model, or the number of events within the dataset was not specified. The median (Interquartile-range (IQR)) event rate was summarised. Results relating to this PROBAST item can be found in Table 2.

Were continuous and categorical predictions handled appropriately? (PROBAST 4.2)

Studies which use categorisation when analysing continuous predictors are usually rated as high risk of bias in the PROBAST assessment, unless a clear clinical rationale is provided for doing so [9]. Results relating to this PROBAST item can be found in Table 3.

Were all enrolled participants included in the analysis? (PROBAST 4.3)

The PROBAST assessment includes determining if all enrolled participants were included in the analysis, and if a study excluded participants, the reason for this must be justified for doing so [9]. Results relating to this PROBAST item can be found in Table 4.

Were participants with missing data handled appropriately? (PROBAST 4.4)

The majority of studies, 227 (75.4%), did not adequately report a specific approach for handling missing data for either the outcome or covariates. Where this was reported, the type of methods used to handle missing data can be found in Table 5. Some studies reported more than one method for handling missing data.

Additionally, two (0.7%) studies created an extra category for each variable used in the analysis which had missing data to minimise the loss of observations through missing data. One (0.3%) study excluded variables if more than 10% of the data for that variable was missing and one (0.3%) study only used variables in the analysis which had fewer than 20% missing data.

Was selection of predictors based on univariable analysis avoided? (PROBAST 4.5)

Univariable screening, use of stepwise regression (for example, backwards or forwards elimination) when choosing predictors for inclusion in the final model are characteristics associated with high risk of bias according to PROBAST [9]. The number of included studies which reported using these can be found in Table 6.

Were complexities in the data accounted for appropriately? (PROBAST 4.6)

This section of the PROBAST domain was not summarised, as recurrent event data is already considered a complexity. Therefore, all included papers could be classified as accounting for complexities in the data.

*Were relevant model performance measures evaluated appropriately? (PROBAST 4.7)*

The PROBAST checklist requires internal validation and reporting of calibration and discrimination statistics for a study to be rated as a low risk of bias [9]. Some papers used multiple measures of internal validation, where several measures for calibration and discrimination were reported. External validation was found to be used far less, in only three (1.0%) of included studies [16, 36, 37], although notably models may have been externally validated in separate publications that were not picked up by our review. Results relating to this PROBAST item can be found in Table 7.

*Were model overfitting, underfitting, and optimism in model performance accounted for? (PROBAST 4.8)*

Following internal validation, studies which account for model overfitting and optimism model are graded as low risk of bias according to PROBAST [9]. Results relating to this PROBAST item can be found in in Table 8.

*Do predictors and their assigned weights in the final model correspond to the results from the reported multivariable model? (PROBAST 4.9)*

To be graded as low risk of bias according to PROBAST [9], studies should report all the predictors included in the final model and levels for each. Studies should also report the full results for all included predictors. Results relating to this PROBAST item can be found in Table 9.

### Additional information

Few studies calculated additional statistics to assess model performance and model fit which are currently outside the scope of PROBAST. These results can be found in Table 10.

**Table 10:** Additional information

Model performance statistics	Number (%) of included studies
Root mean square error (RMSE)	7 (2.3%)
Mean absolute percentage error (MAPE)	1 (0.3%)
Mean square error (MSE)	2 (0.7%)
Root mean square percentage error (RMSPE)	0 (0.0%)
Akaike information criterion (AIC) statistic	23 (7.6%)
Bayesian information criterion (BIC) statistic	9 (3.0%)
Deviance information criterion (DIC) statistic	7 (2.3%)

## Discussion

This systematic review demonstrated that a wide range of statistical methods are used in practice when developing prediction models for recurrent event data. There were 11 methods identified in total to analyse recurrent events. The most commonly applied method was the Andersen-Gill model and cardiology was the most frequently reported clinical area. Many studies were rated as high risk of bias according to the analysis domain of the PROBAST assessment tool, primarily due to a lack of (internal) validation and a lack of reporting of performance measures by only reporting the effect size, 95% CI and *p* value in the results. Model overfitting/underfitting was also poorly examined. High risk of bias was also identified where studies did not fully report the results in the paper by not including the estimates for all predictors included in the model. How predictors were chosen for model inclusion also indicated a risk of bias through the use of univariable screening, and the dichotomised of continuous variables was also seen. Key items were also missing in some of the papers, such as the number of patients who experienced events, the total number of recurrent events, the length of follow-up or the number of predictors in the model. This resulted in the event rate or EPV being unable to be calculated for all studies, and for the ones where it could a high risk of bias was identified for some papers here also.

To the best of our knowledge, this is the first systematic review of prediction models which focuses solely on the methodology used to analyse recurrent event data rather than a specific clinical area or study setting. The largest systematic review of prediction models to date is a review of prediction models for diagnosis and prognosis of COVID-19 [38]. This highlighted that almost all published prediction models were poorly reported and at high risk of bias such that their reported predictive performance is likely to be optimistic. These findings are in line with our systematic review. An additional systematic review on recurrent events was conducted, but it is specific to interventions to prevent recurrent falls published in 2009 which includes papers published until 2006 [39].

There are limitations to this review, namely that a single database was searched, and in 2019. However, an extensive and diverse range of models was identified from MEDLINE alone which we feel reflects findings that would be obtained from additional databases and more research running of the search strategy. Statistical practice has changed very little in the last five years with regards to modelling of recurrent events. Therefore, it is unlikely that the main results would change if a more recent search had been run. In addition, only 301 papers met the pre-specified inclusion/exclusion criteria, despite no limit being placed on factors such as clinical area, study period and population. Therefore, it is also unlikely that searching of an additional database such as EMBASE would result in a substantial number of additional papers. Also, some studies did not report certain information. It may have been possible to obtain this additional information by contacting the corresponding author. However, the purpose of this review is to identify methods for modelling recurrent events and not to undertake a full quality assessment and therefore this was felt to be unnecessary for this review. A final limitation is that names of methods known prior to conducting the review to analyse recurrent events were included in the search strategy, which may have caused bias in the search results. However, to the best of our knowledge, all methods available to analyse recurrent events are included in our strategy.

The variability of the approaches identified suggests a lack of knowledge and expertise in the field, highlighting the need for more methodological research to bring greater consistency in the approach to recurrent event analysis. Furthermore, when assessing papers for inclusion in review, there were examples identified which handled the recurrent event data inappropriately and were thus excluded. For example, deriving a binary variable which captured whether patients experienced recurrences, which was then analysed using logistic regression rather than utilising a recurrent event analysis method [40–53]. This indicates a further lack of knowledge in the field of recurrent event analysis amongst researchers, and therefore a need to provide evidence and inform researchers of methods available.

This review identified a number of statistical methods for modelling recurrent event data. There is therefore a need to identify whether models are suited to a particular clinical scenario, or whether they can be used interchangeably. In addition, research is required regarding which summary measures can be used to differentiate between prediction models for recurrent events, for example to summarise their predictive performance. Further work is required in this area to encourage the development and validation of statistically robust prediction models, and the appropriate reporting of prediction models via the pre-existing transparent reporting of a multivariable prediction model for individual prognosis or diagnosis (TRIPOD) reporting guidelines [54]. This will ensure that prediction models that are adopted within clinical practice are robust and appropriate to the clinical setting, including modelling all events along a patient’s journey, not just the first.

## Conclusions

This systematic review identified a wide range of statistical methods that have been applied in practice to develop and validate prediction models with recurrent event data. The Andersen-Gill model was found to be the most frequently applied. The review also identified several types of frailty models which can be used to analyse recurrent events. The results of the systematic review and the variety of methods identified highlight the need for further methodological research to bring greater consistency in the analysis methods used for recurrent event analysis.

Very few studies performed any type of model validation and reporting of model performance statistics was rare. Further work is now required to determine which, if any, models may be better suited to analyse recurrent events under different scenarios. Additional work is also required to support authors to develop and validate robust statistical models, and report them appropriately according to the TRIPOD statement. 

## Data Availability

Not applicable.

## Appendix 1

Search strategy

**Table 11:** Search strategy used for review

No	Search Term	Field
1	Predict*	All Fields
2	Model*	All Fields
3	Prognos*	All Fields
4	Risk	All Fields
5	Analy$ing	All Fields
6	Analy$e	All Fields
7	Survival	All Fields
8	OR 1–7	
9	Repeat*	All Fields
10	Recur*	All Fields
11	Multiple Failure	All Fields
12	Multiple Relapse	All Fields
13	‘Multiple time-to-event’	All Fields
14	OR 9–13	
15	8 AND 14	
16	Exten* adj3 Cox	All Fields
17	Frailty	All Fields
18	Multi State Model	All Fields
19	OR 16–18	
20	15 AND 19	
21	Andersen Gill	All Fields
22	Anderson Gill	All Fields
23	Prentice Williams Peterson	All Fields
24	PWP-TT	All Fields
25	PWP-GT	All Fields
26	PWP-CP	All Fields
27	Wei Lin Weissfeld	All Fields
28	WLW	All Fields
29	‘Proportional Intensity model’	All Fields
30	OR 21–29	
31	20 OR 30	

^*^represents wildcard terms to retrieve variants of the phrase listed, and $ is used to detect both British and American spellings

## Appendix 2

**Table 12:** List of included studies

Paper	Reference
1	Aalen OO, Fosen J, Weedon-Fekjaer H, Borgan ÿ, Husebye E. Dynamic Analysis of Multivariate Failure Time Data. Biometrics. 2004;60(3):764–73
2	Abbai NS, Nyirenda M, Naidoo S, Ramjee G. Prevalent Herpes Simplex Virus-2 Increases the Risk of Incident Bacterial Vaginosis in Women from South Africa. AIDS and Behavior. 2017;22(7):2172–80
3	Abu-Zeitone A, Peterson DR, Polonsky B, McNitt S, Moss AJ. Oral contraceptive use and the risk of cardiac events in patients with long QT syndrome. Heart Rhythm. 2014;11(7):1170–5
4	Adachi JD, Berger C, Barron R. Predictors of imminent non-vertebral fracture in elderly women with osteoporosis, low bone mass, or a history of fracture, based on data from the population-based Canadian Multicentre Osteoporosis Study (CaMos). Archives of Osteoporosis. 2019;14(1)
5	Aleixo ALQdC, Vasconcelos C. de Oliveira R, Cavalcanti Albuquerque M, Biancardi AL, Land Curi AL, Israel Benchimol E, et al. Toxoplasmic retinochoroiditis: The influence of age, number of retinochoroidal lesions and genetic polymorphism for IFN-? + 874 T/A as risk factors for recurrence in a survival analysis. Plos One. 2019;14(2):e0211627
6	Amorim LDAF, Cai J. Modelling recurrent events: a tutorial for analysis in epidemiology. International Journal of Epidemiology. 2014;44(1):324–33
7	Anguita S·nchez M, Bertomeu MartÌnez V, Ruiz Ortiz M, et al. Direct oral anticoagulants versus vitamin K antagonists in real-world patients with nonvalvular atrial fibrillation. The FANTASIIA study. Revista EspaÒola de CardiologÌa (English Edition). 2020;73(1):14–20
8	Arintaya Phrommintikul A, Chinwong S, Patumanond J, Chinwong D, Joseph Hall J. Reduction in total recurrent cardiovascular events in acute coronary syndrome patients with low-density lipoprotein cholesterol goal <70 mg/dL: a real-life cohort in a developing country. Therapeutics and Clinical Risk Management. 2016:353
9	Asada S, Yoshida K, Fukuma S, et al. Effectiveness of cinacalcet treatment for secondary hyperparathyroidism on hospitalization: Results from the MBD-5D study. Plos One. 2019;14(5):e0216399
10	Bachmann JM, Duncan MS, Shah AS, et al. Association of Cardiac Rehabilitation With Decreased Hospitalizations and Mortality After Ventricular Assist Device Implantation. JACC: Heart Failure. 2018;6(2):130–9
11	Baeten JM, Nyange PM, Richardson BA, Lavreys L, Chohan B, Martin HL, et al. Hormonal contraception and risk of sexually transmitted disease acquisition: Results from a prospective study. American Journal of Obstetrics and Gynecology. 2001;185(2):380–5
12	Bagnasco F, Haupt R, Fontana V, Valsecchi MG, Rebora P, Caviglia I, et al. Risk of repeated febrile episodes during chemotherapy-induced granulocytopenia in children with cancer: a prospective single center study. Journal of Chemotherapy. 2012;24(3):155–60
13	Balan T-A, Boonk SE, Vermeer MH, Putter H. Score test for association between recurrent events and a terminal event. Statistics in Medicine. 2016;35(18):3037–48
14	Balan TA, Jonker MA, Johannesma PC, Putter H. Ascertainment correction in frailty models for recurrent events data. Statistics in Medicine. 2016;35(23):4183–201
15	Balkus JE, Jaoko W, Mandaliya K, et al. The Posttrial Effect of Oral Periodic Presumptive Treatment for Vaginal Infections on the Incidence of Bacterial Vaginosis and Lactobacillus Colonization. Sexually Transmitted Diseases. 2012;39(5):361–5
16	BarcelÛ MA, RodrÌguez-Poncelas A, Saez M, Coll-de-Tuero G. The dynamic behaviour of metabolic syndrome and its components in an eight-year population-based cohort from the Mediterranean. Plos One. 2017;12(5):e0176665
17	Bautista CT, Wurapa EK, Sanchez JL. Does the Hazard of Chlamydia Increase with the Number of Gonorrhea Diagnoses? A Large Population-Based Study Among U.S. Army Women. Journal of Women's Health. 2019;28(2):220–4
18	Bautista CT, Wurapa EK, Sateren WB, Hollingsworth BP, Sanchez JL. Longitudinal association of gonorrhea and bacterial vaginosis with repeat chlamydia diagnoses among U.S. Army women: a retrospective cohort analysis. Military Medical Research. 2018;5(1)
19	Beachler DC, D'Souza G, Sugar EA, Xiao W, Gillison ML. Natural History of Anal vs Oral HPV Infection in HIV-Infected Men and Women. The Journal of Infectious Diseases. 2013;208(2):330–9
20	Beachler DC, Sugar EA, Margolick JB, et al. Risk Factors for Acquisition and Clearance of Oral Human Papillomavirus Infection Among HIV-Infected and HIV-Uninfected Adults. American Journal of Epidemiology. 2014;181(1):40–53
21	Beachler DC, Viscidi R, Sugar EAa, et al. A Longitudinal Study of Human Papillomavirus 16 L1, E6, and E7 Seropositivity and Oral Human Papillomavirus 16 Infection. Sexually Transmitted Diseases. 2015;42(2):93–7
22	Benavides FG. Occupational categories and sickness absence certified as attributable to common diseases. The European Journal of Public Health. 2003;13(1):51–5
23	Benavides FG, S·ez M, BarcelÛ MA, Serra C, Mira M. Temporary disability: analysis strategies. Gac Sanit. 1999;13(3):185–90
24	Benavides FG, Tor· I, Miguel MartÌnez J, JardÌ J, Manzanera R, Alberti C, et al. EvaluaciÛn de la gestiÛn de los casos de incapacidad temporal por contingencia com˙n de m·s de 15 dÌas en CataluÒa. Gaceta Sanitaria. 2010;24(3):215–9
25	Bhatt DL, Steg PG, Miller M, et al. Effects of Icosapent Ethyl on Total†Ischemic Events. Journal of the American College of Cardiology. 2019;73(22):2791–802
26	Bonandera C, Jernbroa C. Does gender moderate the association between intellectual ability and accidental injuries? Evidence from the 1953 Stockholm Birth Cohort study. Elsevier: Science direct. 2017
27	Bouaziz O, Courtin D, Cottrell G, Milet J, Nuel G, Garcia A. Is Placental Malaria a Long-term Risk Factor for Mild Malaria Attack in Infancy? Revisiting a Paradigm. Clinical Infectious Diseases. 2017;66(6):930–5
28	Braga JR, Tu JV, Austin PC, Sutradhar R, Ross HJ, Lee DS. Recurrent events analysis for examination of hospitalizations in heart failure: insights from the Enhanced Feedback for Effective Cardiac Treatment (EFFECT) trial. European Heart Journal—Quality of Care and Clinical Outcomes. 2017;4(1):18–26
29	Brand„o TB, Vechiato Filho AJ, de Souza Batista VE, Prado Ribeiro AC, Filho HN, Chilvarquer I, et al. Assessment of treatment outcomes for facial prostheses in patients with craniofacial defects: A pilot retrospective study. The Journal of Prosthetic Dentistry. 2017;118(2):235–41
30	Capodanno D, Gargiulo G, Buccheri S, et al. Computing Methods for Composite Clinical Endpoints in Unprotected Left Main Coronary Artery Revascularization. Journal of American College Cardiology. 2016;9(22)
31	Chamberlain AM, Alonso A, Gersh BJ, et al. Multimorbidity and the risk of hospitalization and death in atrial fibrillation: A population-based study. American Heart Journal. 2017;185:74–84
32	Chamberlain AM, Rutten LJF, Jacobson DJ. Multimorbidity, functional limitations, and outcomes: Interactions in a population-based cohort of older adults. Journal of Comorbidity. 2019;9:2235042X1987348
33	Charles-Nelson A, Katsahian S, Schramm C. How to analyze and interpret recurrent events data in the presence of a terminal event: An application on readmission after colorectal cancer surgery. Statistics in Medicine. 2019
34	Chaudhry SI, McAvay G, Chen S, Whitson H, Newman AB, Krumholz HM, et al. Risk Factors for Hospital Admission Among Older Persons With Newly Diagnosed Heart Failure. Journal of the American College of Cardiology. 2013;61(6):635–42
35	Cheek SM, Goldston DB, Erkanli A, Massing-Schaffer M, Liu RT. Social Rejection and Suicidal Ideation and Attempts among Adolescents Following Hospitalization: a Prospective Study. Research on Child and Adolescent Psychopathology. 2019;48(1):123–33
36	Chen C-M, Shen P-s. Semiparametric regression analysis of failure time data with dependent interval censoring. Statistics in Medicine. 2017;36(21):3398–411
37	Chen M-H, Ibrahim JG, Zeng D, Hu K, Jia C. Bayesian design of superiority clinical trials for recurrent events data with applications to bleeding and transfusion events in myelodyplastic syndrome. Biometrics. 2014;70(4):1003–13
38	Chiou SH, Xu G, Yan J, Huang C-Y. Semiparametric estimation of the accelerated mean model with panel count data under informative examination times. Biometrics. 2017;74(3):944–53
39	Cousins Gi, Boland F, Barry J, et al. J-shaped relationship between supervised methadone consumption and retention in methadone maintenance treatment (MMT) in primary care: National cohort study. Elsevier. 2017;173:126–31
40	Cowie MR, Sarkar S, Koehler J, Whellan DJ, Crossley GH, Tang WHW, et al. Development and validation of an integrated diagnostic algorithm derived from parameters monitored in implantable devices for identifying patients at risk for heart failure hospitalization in an ambulatory setting. European Heart Journal. 2013;34(31):2472–80
41	Cui J, Forbes A, Kirby A, Marschner I, Simes J, Hunt D, et al. Semi-parametric risk prediction models for recurrent cardiovascular events in the LIPID study. BMC Medical Research Methodology. 2010;10(1)
42	Cui J, Forbes A, Kirby A, Marschner I, Simes J, West M, et al. Parametric conditional frailty models for recurrent cardiovascular events in the lipid study. Clinical Trials. 2008;5(6):565–74
43	Dagne GA, Snyder J. Bayesian Analysis of Repeated Events Using Event-Dependent Frailty Models: An Application to Behavioral Observation Data. Communications in Statistics—Theory and Methods. 2009;39(2):293–310
44	Dalrymple LS, Johansen KL, Chertow GM, Cheng S-C, Grimes B, Gold EB, et al. Infection-Related Hospitalizations in Older Patients With ESRD. American Journal of Kidney Diseases. 2010;56(3):522–30
45	Damon W, McNeil R, Milloy MJ, Nosova E, Kerr T, Hayashi K. Residential eviction predicts initiation of or relapse into crystal methamphetamine use among people who inject drugs: a prospective cohort study. Journal of Public Health. 2019;41(1):36–45
46	de Carpentier JP, Ryder WDJ, Saeed SR, Woolford TJ. Survival times of Provoxô valves. The Journal of Laryngology & Otology. 1996;110(1):37–42
47	Di Mario S, Gagliotti C, Donatini A, et al. Formula feeding increases the risk of antibiotic prescriptions in children up to 2†years: results from a cohort study. European Journal of Pediatrics. 2019;178(12):1867–74
48	Di Napoli A, Pezzotti P, Di Lallo D, Tancioni V, Papini P, Guasticchi G. Determinants of hospitalization in a cohort of chronic dialysis patients in central Italy. J Nephrol 2005;. 2005;18(1):21–9
49	DiabatÈ S, Chamberland A, Geraldo N, Tremblay C, Alary M. Gonorrhea, Chlamydia and HIV incidence among female sex workers in Cotonou, Benin: A longitudinal study. Plos One. 2018;13(5):e0197251
50	Diel IJ, Weide R, Kˆppler H, Antr‡s L, Smith M, Green J, et al. Risk of renal impairment after treatment with ibandronate versus zoledronic acid: a retrospective medical records review. Supportive Care in Cancer. 2008;17(6):719–25
51	Donio PJ, Freitas C, Austin PC, et al. Comparison of Readmission and Death Among Patients With Cardiac Disease in Northern vs Southern Ontario. Canadian Journal of Cardiology. 2019;35(3):341–51
52	Doran MF, Crowson CS, Pond GR, O'Fallon WM, Gabriel SE. Frequency of infection in patients with rheumatoid arthritis compared with controls: A population-based study. Arthritis & Rheumatism. 2002;46(9):2287–93
53	Du P, Jiang Y, Wang Y. Smoothing Spline ANOVA Frailty Model for Recurrent Event Data. Biometrics. 2011;67(4):1330–9
54	Dunlay SM, Manemann SM, Chamberlain AM, Cheville AL, Jiang R, Weston SA, et al. Activities of Daily Living and Outcomes in Heart Failure. Circulation: Heart Failure. 2015;8(2):261–7
55	Dunlay SM, Redfield MM, Weston SA, Therneau TM, Hall Long K, Shah ND, et al. Hospitalizations After Heart Failure Diagnosis. Journal of the American College of Cardiology. 2009;54(18):1695–702
56	Dunlay SM, Roger VL, Weston SA, Bangerter LR, Killian JM, Griffin JM. Patient and Spousal Health and Outcomes in Heart Failure. Circulation: Heart Failure. 2017;10(10)
57	Dunn C, Emeasoba EU, Hung M, Holtzman A, Bellin E, Greenstein S. A Retrospective Cohort Study on Rehospitalization following Expanded Criteria Donor Kidney Transplantation. Surgery Research and Practice. 2018;2018:1–8
58	Esber A, Polyak C, Kiweewa F, et al. Persistent Low-level Viremia Predicts Subsequent Virologic Failure: Is It Time to Change the Third 90? Clinical Infectious Diseases. 2018;69(5):805–12
59	Espel JC, Palac HL, Cullina JF, et al. Antibiotic duration and changes in FEV1 are not associated with time until next exacerbation in adult cystic fibrosis: a single center study. BMC Pulmonary Medicine. 2017;17(1)
60	Eythorsson E, Sigurdsson S, Hrafnkelsson B, ErlendsdÛttir H, Haraldsson s, Kristinsson KG. Impact of the 10-valent pneumococcal conjugate vaccine on antimicrobial prescriptions in young children: a whole population study. BMC Infectious Diseases. 2018;18(1)
61	Fabbri M, Yost K, Finney Rutten LJ, et al. Health Literacy and Outcomes in Patients With Heart Failure: A Prospective Community Study. Mayo Clinic Proceedings. 2018;93(1):9–15
62	Figueiras A, Lado E, Fern·ndez S, Hervada X. Influence of physicians' attitudes on under-notifying infectious diseases: a longitudinal study. Public Health. 2004;118(7):521–6
63	Flohil SC, De Vries E, Van Meurs JBJ, Fang Y, Stricker BHC, Uitterlinden AG, et al. Vitamin D-binding protein polymorphisms are not associated with development of (multiple) basal cell carcinomas. Experimental Dermatology. 2010;19(12):1103–5
64	Frick U, Frick H, Langguth B, Landgrebe M, H¸bner-Liebermann B, Hajak G. The Revolving Door Phenomenon Revisited: Time to Readmission in 17í415 Patients with 37í697 Hospitalisations at a German Psychiatric Hospital. PLoS ONE. 2013;8(10):e75612
65	Fu H, Luo J, Qu Y. Hypoglycemic events analysis via recurrent time-to-event (HEART) models. Journal of Biopharmaceutical Statistics. 2016;26(2):280–98
66	Gabbett TJ, Ullah S. Relationship between running loads and soft-tissue injury in elite team sport athletes. Journal of Strength and Conditioning Research. 2012;26(4):953–60
67	Gabbett TJ, Ullah S, Finch CF. Identifying risk factors for contact injury in professional rugby league players ñ Application of a frailty model for recurrent injury. Journal of Science and Medicine in Sport. 2012;15(6):496–504
68	Gao S, Zhou X–H. An empirical comparison of two semi-parametric approaches for the estimation of covariate effects from multivariate failure time data. Statistics in Medicine. 1997;16(18):2049–62
69	Genser B, Wernecke KD. Joint Modelling of Repeated Transitions in Follow-up Data—A Case Study on Breast Cancer Data. Biometrical Journal. 2005;47(3):388–401
70	Giha H. The epidemiology of febrile malaria episodes in an area of unstable and seasonal transmission. Transactions of the Royal Society of Tropical Medicine and Hygiene. 2000;94(6):645–51
71	Gill TM, Guo Z, Allore HG. The Epidemiology of Bathing Disability in Older Persons. Journal of the American Geriatrics Society. 2006;54(10):1524–30
72	Gisev N, Bharat C, Larney S, et al. The effect of entry and retention in opioid agonist treatment on contact with the criminal justice system among opioid-dependent people: a retrospective cohort study. The Lancet Public Health. 2019;4(1):e334-42
73	Gohari MR, Khodabakhshi R, Shahidi J. The impact of multiple recurrences in disease-free survival of breast cancer: an extended Cox model. Biblioteca Istituto Tumori. 2012;98:428–33
74	Gohari MR, Mahmoudi M, Mohammed K, Pasha E, Khodabakhshi R. Recurrence in breast cancer. Analysis with frailty model. Saudi Med Journal. 2006;27(8):1187–93
75	Goldenberg I, Hall WJ, Beck CA, Moss AJ, Barsheshet A, McNitt S, et al. Reduction of the Risk of Recurring Heart Failure Events With Cardiac Resynchronization Therapy. Journal of the American College of Cardiology. 2011;58(7):729–37
76	Goldman-Hasbun J, Nosova E, Kerr T, Wood E, DeBeck K. Homelessness and incarceration associated with relapse into stimulant and opioid use among youth who are street-involved in Vancouver, Canada. Drug and Alcohol Review. 2019;38(4):428–34
77	Goodday SM, Preisig M, Gholamrezaee M, Grof P, Duffy A. Temperament and self-esteem in high-risk offspring of bipolar parents: Vulnerability and scar effects. Journal of Affective Disorders. 2019;243:209–15
78	Goodman AC, Hankin JR, Kalist DE, Peng Y, Spurr SJ. Estimating Determinants of Multiple Treatment Episodes for Substance Abusers. The Journal of Mental Health Policy and Economics J Mental Health Policy Econ. 2001;4:65–77
79	Grimaldi-Bensouda L, Zuerik M, Aubier M, et al. Does omalizumab make a difference to the real-life treatment of asthma exacerbations?: Results from a large cohort of patients with severe uncontrolled asthma. CHEST Journal. 2013;143(2):398—405
80	Gronefeld GC, Israel CW, Padmanabhan V, Koehler J, Cuijpers A, Hohnloser SH. Ventricular Rate Stabilization for the Prevention of Pause Dependent Ventricular Tachyarrhythmias: Results from a Prospective Study in 309 ICD Recipients. Pacing and Clinical Electrophysiology. 2002;25(12):1708–14
81	Gruneir A, Cigsar C, Wang X, et al. Repeat emergency department visits by nursing home residents: a cohort study using health administrative data. BMC Geriatrics. 2018;18(1)
82	GuÈdou FA, Van Damme L, Deese J, Crucitti T, Becker M, Mirembe F, et al. Behavioural and medical predictors of bacterial vaginosis recurrence among female sex workers: longitudinal analysis from a randomized controlled trial. BMC Infectious Diseases. 2013;13(1)
83	Guo Z, Gill TM, Allore HG. Modeling repeated time-to-event health conditions with discontinuous risk intervals: an example of a longitudinal study of functional disability among older persons. Methods Inf Med. 2008;47(2):107ñ16
84	Haddad L, Wall KM, Vwalika B, et al. Contraceptive discontinuation and switching among couples receiving integrated HIV and family planning services in Lusaka, Zambia. Aids. 2013;27(Supplement 1):S93-S103
85	Haddad LB, Wall KM, Tote K, et al. Hormonal Contraception and Vaginal Infections Among Couples Who Are Human Immunodeficiency Virus Serodiscordant in Lusaka, Zambia. Obstetrics & Gynecology. 2019;134(3):573–80
86	Hartog LC, Cimzar-Sweelssen M, Knipscheer A, Groenier KH, Kleefstra N, Bilo HJG, et al. Orthostatic hypotension does not predict recurrent falling in a nursing home population. Elsevier: Archives of Gerontology and Geriatrics. 2017;68:39ñ43
87	Hasin T, Marmor Y, Kremers W, et al. Readmissions After Implantation of Axial Flow Left Ventricular Assist Device. Journal of the American College of Cardiology. 2013;61(2):153–63
88	Hawkins AT, Schaumeier MJ, Smith AD, Hevelone ND, Nguyen LL. When to Call it a Day: Incremental Risk of Amputation and Death after Multiple Revascularization. Annals of Vascular Surgery. 2014;28(1):35–47
89	He X, Tong X, Sun J. Semiparametric analysis of panel count data with correlated observation and follow-up times. Lifetime Data Analysis. 2008;15(2):177–96
90	HervÈ D, Ibos-AugÈ N, CalviËre L, et al. Predictors of clinical or cerebral lesion progression in adult moyamoya angiopathy. Neurology. 2019;93(4):e388-e97
91	Heussen N, Hilgers R-D, Heimann H, Collins L, Grisanti S. Scleral buckling versus primary vitrectomy in rhegmatogenous retinal detachment study (SPR Study): Multiple-event analysis of risk factors for reoperations. SPR Study report no. 4. Acta Ophthalmologica. 2009;89(7):622–8
92	Holland GN, Crespi CM, ten Dam-van Loon N, Charonis AC, Yu F, Bosch-Driessen LH, et al. Analysis of Recurrence Patterns Associated with Toxoplasmic Retinochoroiditis. American Journal of Ophthalmology. 2008;145(6):1007–13.e1
93	Hosseini S, Moghimbeigi A, Roshanaei G, Momeniarbat F. Evaluation of Drug Abuse Relapse Event Rate Over Time in Frailty Model. Osong Public Health and Research Perspectives. 2014;5(2):92–5
94	Huang C-Y, Wang M-C. Joint Modeling and Estimation for Recurrent Event Processes and Failure Time Data. Journal of the American Statistical Association. 2004;99(468):1153–65
95	Huang CY, Qin J, Wang MC. Semiparametric Analysis for Recurrent Event Data with Time-Dependent Covariates and Informative Censoring. Biometrics. 2009;66(1):39–49
96	Hughes J, Jokubaitis V, Lugaresi A, et al. Association of Inflammation and Disability Accrual in Patients With Progressive-Onset Multiple Sclerosis. JAMA Neurology. 2018;75(11):1407
97	Hunter LC, Lee RJ, Butcher I, et al. Patient characteristics associated with risk of first hospital admission and readmission for acute exacerbation of chronic obstructive pulmonary disease (COPD) following primary care COPD diagnosis: a cohort study using linked electronic patient records. BMJ Open. 2016;6(1):e009121
98	Jensen AKG, Ravn H, S¯rup S, Andersen P. A marginal structural model for recurrent events in the presence of time-dependent confounding: non-specific effects of vaccines on child hospitalisations. Statistics in Medicine. 2016;35(27):5051–69
99	Jeon-Slaughter H, Tsai S, Kamath P, et al. Comparison of Lower Extremity Endovascular Intervention Outcomes in Women Versus Men. Elsevier. 2016
100	Jones CL, Harrington DP. Omnibus Tests of The Martingale Assumption in The Analysis of Recurrent Failure Time Data. Lifetime Data Analysis. 2001;7(1):157ñ71
101	Juarez-Colunga E, Rosenfeld M, Zemanick ET, Wagner B. Application of multiple event analysis as an alternative approach to studying pulmonary exacerbations as an outcome measure. Journal of Cystic Fibrosis. 2020;19(1):114–8
102	Jung TH, Peduzzi P, Allore H, Kyriakides TC, Esserman D. A joint model for recurrent events and a semi-competing risk in the presence of multi-level clustering. Statistical Methods in Medical Research. 2018;28(10–11):2897–911
103	Kalincik T, Vivek V, Jokubaitis V, et al. Sex as a determinant of relapse incidence and progressive course of multiple sclerosis. Brain. 2013;136(12):3609–17
104	Kamakura T, Yanagimoto T. Point process models in asthma attacks for assessing environmental risk factors. Environmental Health Perspectives. 1985;63:203–10
105	Kemner SM, Mesman E, Nolen WA, Eijckemans MJC, Hillegers MHJ. The role of life events and psychological factors in the onset of first and recurrent mood episodes in bipolar offspring: results from the Dutch Bipolar Offspring Study. Psychological Medicine. 2015;45(12):2571–81
106	Kemner SM, van Haren NEM, Bootsman F, Eijkemans MJC, Vonk R, van der Schot AC, et al. The influence of life events on first and recurrent admissions in bipolar disorder. International Journal of Bipolar Disorders. 2015;3(1)
107	Kennedy MC, Klassen DC, Dong H, Milloy MJS, Hayashi K, Kerr TH. Supervised Injection Facility Utilization Patterns: A Prospective Cohort Study in Vancouver, Canada. American Journal of Preventive Medicine. 2019;57(3):330–7
108	Kessing LV, Hansen MG, Andersen PK, Angst J. The predictive effect of episodes on the risk of recurrence in depressive and bipolar disorders—a life-long perspective. Acta Psychiatrica Scandinavica. 2004;109(5):339–44
109	Kessing LV, Olsen EW, Andersen PK. Recurrence in Affective Disorder: Analyses with Frailty Models. American Journal of Epidemiology. 1999;149(5):404–11
110	Kheiri S, Alibeigi Z. An analysis of first-time blood donors return behaviour using regression models. Transfusion Medicine. 2015;25(4):243–8
111	Kiburg KV, Ward GM, Vogrin S, Steele K, Mulrooney E, Loh M, et al. Impact of type 2 diabetes on hospitalization and mortality in people with malignancy. Diabetic Medicine. 2019;37(2):362–8
112	Kiiski V, de Vries E, Flohil SC, Bijl MJ, Hofman A, Stricker BHC, et al. Risk Factors for Single and Multiple Basal Cell Carcinomas. Archives of Dermatology. 2010;146(8)
113	Kim S-M, Park J, Kim SH, Park S-Y, Kim JY, Sung J-J, et al. Factors Associated With the Time to Next Attack in Neuromyelitis Optica: Accelerated Failure Time Models With Random Effects. PLoS ONE. 2013;8(12):e82325
114	Koren A, Steinberg DM, Drory Y, Gerber Y. Socioeconomic environment and recurrent coronary events after initial myocardial infarction. Annals of Epidemiology. 2012;22(8):541–6
115	Koton S, Molshatzki N, Yuval, Myers V, Broday DM, Drory Y, et al. Cumulative exposure to particulate matter air pollution and long-term post-myocardial infarction outcomes. Preventive Medicine. 2013;57(4):339–44
116	Kougias P, Sharath S, Barshes NR, Lowery B, Garcia A, Pak T, et al. Impact of cumulative intravascular contrast exposure on renal function in patients with occlusive and aneurysmal vascular disease. Journal of Vascular Surgery. 2014;59(6):1644–50
117	KrÛl A, Tournigand C, Michiels S, Rondeau V. Multivariate joint frailty model for the analysis of nonlinear tumor kinetics and dynamic predictions of death. Statistics in Medicine. 2018;37(13):2148–61
118	Kubo J, Goldstein BA, Cantley LF, Tessier-Sherman B, Galusha D, Slade MD, et al. Contribution of health status and prevalent chronic disease to individual risk for workplace injury in the manufacturing environment. Occupational and Environmental Medicine. 2013;71(3):159–66
119	Ladak F, Socias E, Nolan S, et al. Substance use Patterns and HIV-1 RNA Viral Load Rebound among HIV-Positive Illicit Drug users in a Canadian Setting. Antiviral Therapy. 2019;24(1):19–25
120	Lafata JE, Pladevall M, Divine G, Ayoub M, Philbin EF. Are There Race/Ethnicity Differences in Outpatient Congestive Heart Failure Management, Hospital Use, and Mortality Among an Insured Population? Medical Care. 2004;42(7):680–9
121	Lam JO, Bream JH, Sugar EA, et al. Association of serum cytokines with oral HPV clearance. Cytokine. 2016;83:85–91
122	Lam JO, Sugar EA, Cranston RD, et al. The association of medication use with clearance or persistence of oral HPV infection. Cancer Causes & Control. 2016;27(12):1491–8
123	Larsen A, Magasana V, Dinh T-H, et al. Longitudinal adherence to maternal antiretroviral therapy and infant Nevirapine prophylaxis from 6?weeks to 18?months postpartum amongst a cohort of mothers and infants in South Africa. BMC Infectious Diseases. 2019;19(S1)
124	Lavreys L, Chohan V, Overbaugh J, Hassan W, McClelland RS, Kreiss J, et al. Hormonal contraception and risk of cervical infections among HIV-1-seropositive Kenyan women. Aids. 2004;18(16):2179–84
125	Leclerc BS, BÈgin C, Cadieux E, Goulet L, Leduc N, Kergoat MJ, et al. Risk factors for falling among community-dwelling seniors using home-care services: An extended hazards model with time-dependent covariates and multiple events. Chronic Diseases in Canada. 2008;28(4):111–20
126	Lee AH, Gracey M, Wang K, Yau KKW. Under-nutrition Affects Time to Recurrence of Gastroenteritis among Aboriginal and Non-Aboriginal Children. J Health Popul Nutr. 2006;24(1):17–24
127	Lee AH, Wang K, Gracey M, Yau KKW. Factors affecting length of hospitalization of infants and children for recurrent gastroenteritis in Western Australia. Acta Paediatr- Taylor & Francis ISSN 0803–5253. 2003;92(1):843–7
128	Lee CH, Huang C-Y, Xu G, Luo X. Semiparametric regression analysis for alternating recurrent event data. Statistics in Medicine. 2017;37(6):996–1008
129	Lee J, Cook RJ. Dependence modeling for multi-type recurrent events via copulas. Statistics in Medicine. 2019;38(21):4066–82
130	Lee J, Thall PF, Lin SH. Bayesian semiparametric joint regression analysis of recurrent adverse events and survival in esophageal cancer patients. The Annals of Applied Statistics. 2019;13(1)
131	Lee LM, Wortley PM, Fleming PL, Eldred LJ, Gray RH. Duration of Human Immunodeficiency Virus Infection and Likelihood of Giving Birth in a Medicaid Population in Maryland. American Journal of Epidemiology. 2000;151(10):1020–8
132	Lee M, Schwartz J, Wang Y, Dominici F, Zanobetti A. Long-term effect of fine particulate matter on hospitalization with dementia. Environmental Pollution. 2019;254:112,926
133	Lee S, Park H, Kim S, Lee E-K, Lee J, Hong YS, et al. Fine particulate matter and incidence of metabolic syndrome in non-CVD patients: A nationwide population-based cohort study. International Journal of Hygiene and Environmental Health. 2019;222(3):533–40
134	Legrand H, PihlsgÂrd M, Nordell E, ElmstÂhl S. A long-recommended but seldom-used method of analysis for fall injuries found a unique pattern of risk factors in the youngest-old. Aging Clinical and Experimental Research. 2015;27(4):439–45
135	Lemaire M, Islam QS, Shen H, Khan MA, Parveen M, Abedin F, et al. Iron-containing micronutrient powder provided to children with moderate-to-severe malnutrition increases hemoglobin concentrations but not the risk of infectious morbidity: a randomized, double-blind, placebo-controlled, noninferiority safety trial. The American Journal of Clinical Nutrition. 2011;94(2):585–93
136	Liang B, Tong X, Zeng D, Wang Y. Semiparametric regression analysis of repeated current status data. Statistica Sinica. 2017
137	Liang Y, Li Y, Zhang B. Bayesian nonparametric inference for panel count data with an informative observation process. Biometrical Journal. 2018;60(3):583–96
138	Lim HJ, Liu J, Melzer-Lange M. Comparison of Methods for Analyzing Recurrent Events Data: Application to the Emergency Department Visits of Pediatric Firearm Victims. Accident Analysis & Prevention. 2007;39(2):290–9
139	Lin L-A, Luo S, Davis BR. Bayesian regression model for recurrent event data with event-varying covariate effects and event effect. Journal of Applied Statistics. 2017;45(7):1260–76
140	Lipton A, Fizazi K, Stopeck AT, et al. Effect of denosumab versus zoledronic acid in preventing skeletal-related events in patients with bone metastases by baseline characteristics. European Journal of Cancer. 2016;53:75–83
141	Liu B, Lu W, Zhang J. Accelerated intensity frailty model for recurrent events data. Biometrics. 2014;70(3):579–87
142	Liu JF, Jons C, Moss AJ, et al. Risk Factors for Recurrent Syncope and Subsequent Fatal or Near-Fatal Events in Children and Adolescents With Long QT Syndrome. Journal of the American College of Cardiology. 2011;57(8):941–50
143	Liu JF, Moss AJ, Jons C, et al. Mutation-Specific Risk in Two Genetic Forms of Type 3 Long QT Syndrome. The American Journal of Cardiology. 2010;105(2):210–3
144	Liu L, Huang X. The use of Gaussian quadrature for estimation in frailty proportional hazards models. Statistics in Medicine. 2008;27(14):2665–83
145	Liu L, Huang X, Yaroshinsky A, Cormier JN. Joint frailty models for zero-inflated recurrent events in the presence of a terminal event. Biometrics. 2015;72(1):204–14
146	Liu L, Wolfe RA, Huang X. Shared Frailty Models for Recurrent Events and a Terminal Event. Biometrics. 2004;60(3):747–56
147	Livry C, Binquet C, Sgro C, et al. Acute Liver Enzyme Elevations in HIV-1-Infected Patients. HIV Clinical Trials. 2003;4(6):400–10
148	Lombardo-Quezada J, Sanclemente G, Colmenero J, et al. Mannose-Binding Lectin-Deficient Donors Increase the Risk of Bacterial Infection and Bacterial Infection-Related Mortality After Liver Transplantation. American Journal of Transplantation. 2017;18(1):197–206
149	Loureiro MM, Rozenfeld S, Carvalho MS, Portugal RD. Factors associated with hospital readmission in sickle cell disease. BMC Hematology. 2009;9(1)
150	Love A, Kinner S, Young J. Social Environment and Hospitalisation after Release from Prison: A Prospective Cohort Study. International Journal of Environmental Research and Public Health. 2017;14(11):1406
151	Luj·na S, SantamarÌa C, Pontones JL, et al. Risk estimation of multiple recurrence and progression of non-muscle invasive bladder carcinoma using new mathematical models. Elsevier. 2014;38(10):647–54
152	Lyons VH, Kernic MA, Rowhani-Rahbar A, Holt VL, Carone M. Use of multiple failure models in injury epidemiology: a case study of arrest and intimate partner violence recidivism in Seattle, WA. Injury Epidemiology. 2019;6(1)
153	M. Givertz M, W. Stevenson L, R. Costanzo M, et al. Pulmonary Artery Pressure-Guided Management of Patients With Heart Failure and Reduced Ejection Fraction. Journal of the American College Cardiology. 2017;70(15)
154	M. van Striena A, Souverein PC, Keijsersa CJPW, Heerdink ER, Derijks HJ, J. van Marum R. Antipsychotic drug use associated with urinary tract infections inolder women. Elsevier. 2017;Maturitas 98:46ñ50
155	Mador MJ, Choi Y, Bhat A, Dmochowski J, Braun M, Gottumukkala VA, et al. Are the adverse effects of body position in patients with obstructive sleep apnea dependent on sleep stage? Sleep and Breathing. 2009;14(1):13–7
156	MahÈ C, Chevret S. Analysis of recurrent failure times data: should the baseline hazard be stratified. Statistics in Medicine. 2001;20(24):3807–15
157	Maity A, Williams PL, Ryan L, Missmer SA, Coull BA, Hauser R. Analysis of in vitro fertilization data with multiple outcomes using discrete time-to-event analysis. Statistics in Medicine. 2013;33(10):1738–49
158	Malehi AS, Hajizadeh E, Ahmadi K, Kholdi N. Modeling the Recurrent Failure to Thrive in Less than Two-year Children: Recurrent Events Survival Analysis. Journal of Research in Health Sciences. 2014;4(1):97–100
159	Manda SOM, Meyer R. Bayesian inference for recurrent events data using time-dependent frailty. Statistics in Medicine. 2005;24(8):1263–74
160	Manemann SM, Chamberlain AM, Boyd MM, et al. Skilled Nursing Facility Use and Hospitalizations in Heart Failure: A Community Linkage Study. Mayo Clin Proc. 2018
161	Manzini G, Ettrich TJ, Kremer M, et al. Advantages of a multi-state approach in surgical research: how intermediate events and risk factor profile affect the prognosis of a patient with locally advanced rectal cancer. BMC Medical Research Methodology. 2018;18(1)
162	Maradit-Kremers H, Nicola PJ, Crowson CS, OíFallon WM, Gabriel SE. Patient, Disease, and Therapy-Related Factors That Influence Discontinuation of Disease-Modifying Antirheumatic Drugs: A Population-Based Incidence Cohort of Patients with Rheumatoid Arthritis. The Journal of Rheumatology. 2006;33(2)
163	Markt A, Klumpers UMH, Draisma S, et al. Testing a clinical staging model for bipolar disorder using longitudinal life chart data. Bipolar Disorders. 2018;21(3):228–34
164	Maros ME, Schnaidt S, Balla P, et al. In situ cell cycle analysis in giant cell tumor of bone reveals patients with elevated risk of reduced progression-free survival. Bone. 2019;127:188–98
165	Masenyetse LJ, Manda SOM, Mwambi HG. An assessment of adverse drug reactions among HIV positive patients receiving antiretroviral treatment in South Africa. AIDS Research and Therapy. 2015;12(1)
166	Masese L, Baeten JM, Richardson BA, et al. Incidence and Correlates of Chlamydia trachomatis Infection in a High-Risk Cohort of Kenyan Women. Sexually Transmitted Diseases. 2013;40(3):221–5
167	Masoudi S, Ali Montazeri S, Pourdanesh F, et al. A New Approach to Survival Analysis of Head and Neck Squamous Cell Carcinoma. Archives of Iranian Medicine. 2017;Volume 20(Number 8, August 2017):503–9
168	Masri A, Althouse AD, McKibben J, et al. Outcomes of Heart Failure Admissions Under Observation Versus Short Inpatient Stay. Journal of the American Heart Association. 2018;7(3)
169	Matet A, Daruich A, Zola M, Behar-Cohen F. Risk Factors for Recurrences of Central Serous Chorioretinopathy. Retina. 2018;38(7):1403–14
170	Matet A, Paris L, Fardeau C, et al. Clinical and Biological Factors Associated With Recurrences of Severe Toxoplasmic Retinochoroiditis Confirmed by Aqueous Humor Analysis. American Journal of Ophthalmology. 2019;199:82–93
171	Mauguen A, Rachet B, Mathoulin-PÈlissier S, MacGrogan G, Laurent A, Rondeau V. Dynamic prediction of risk of death using history of cancer recurrences in joint frailty models. Statistics in Medicine. 2013;32(30):5366–80
172	Mazroui Y, Mauguen A, Mathoulin-PÈlissier S, MacGrogan G, Brouste V, Rondeau V. Time-varying coefficients in a multivariate frailty model: Application to breast cancer recurrences of several types and death. Lifetime Data Analysis. 2015;22(2):191–215
173	McCarthy JT, Rule AD, Achenbach SJ, Bergstralh EJ, Khosla S, Melton LJ. Use of Renal Function Measurements for Assessing Fracture Risk in Postmenopausal Women. Mayo Clinic Proceedings. 2008;83(11):1231–9
174	McClelland RS, Lavreys L, Katingima C, Overbaugh J, Chohan V, Mandaliya K, et al. Contribution of HIV-1 Infection to Acquisition of Sexually Transmitted Disease: A 10-Year Prospective Study. The Journal of Infectious Diseases. 2005;191(3):333–8
175	McClelland RS, Richardson BA, Graham SM, Masese LN, Gitau R, Lavreys L, et al. A Prospective Study of Risk Factors for Bacterial Vaginosis in HIV-1-Seronegative African Women. Sexually Transmitted Diseases. 2008;35(6):617–23
176	McGilchrist CA, Aisbett CW. Regression with Frailty in Survival Analysis. Biometrics. 1991;47(2):461
177	McNallan SM, Singh M, Chamberlain AM. Frailty and Healthcare Utilization Among Patients With†Heart Failure in†the†Community. JACC: Heart Failure. 2013;1(2):135–41
178	Meister R, Lanio J, Fangmeier T, H‰rter M, Schramm E, Zobel I, et al. Adverse events during a disorder-specific psychotherapy compared to a nonspecific psychotherapy in patients with chronic depression. Journal of Clinical Psychology. 2019;76(1):7–19
179	Melton LJ, Kearns AE, Atkinson EJ, Bolander ME, Achenbach SJ, Huddleston JM, et al. Secular trends in hip fracture incidence and recurrence. Osteoporosis International. 2008;20(5):687–94
180	Melton LJ, Leibson CL, Achenbach SJ, Therneau TM, Khosla S. Fracture Risk in Type 2 Diabetes: Update of a Population-Based Study. Journal of Bone and Mineral Research. 2008;23(8):1334–42
181	Melton LJ, Patel A, Achenbach SJ, Oberg AL, Yunginger JW. Long-Term Fracture Risk Among Children With Asthma: A Population-Based Study. Journal of Bone and Mineral Research. 2004;20(4):564–70
182	Metcalfe C, Thompson SG. Wei, Lin and Weissfeld's marginal analysis of multivariate failure time data. Statistical Methods in Medical Research. 2007;16(2):103–22
183	Mieno MN, Yamaguchi T, Ohashi Y. Alternative statistical methods for estimating efficacy of interferon beta-1b for multiple sclerosis clinical trials. BMC Medical Research Methodology. 2011;11(1)
184	Mogensen UM, Gong J, Jhund PS, et al. Effect of sacubitril/valsartan on recurrent events in the Prospective comparison of ARNI with ACEI to Determine Impact on Global Mortality and morbidity in Heart Failure trial (PARADIGM-HF). European Journal of Heart Failure. 2018;20(4):760–8
185	Molnar AO, Moist L, Klarenbach S, et al. Hospitalizations in Dialysis Patients in Canada: A National Cohort Study. Canadian Journal of Kidney Health and Disease. 2018;5:205,435,811,878,037
186	Monteiro de Castro MS, Carvalho MS, Travassos Cu. Factors associated with readmission to a general hospital in Brazil. Cad Sa˙de P˙blica, Rio de Janeiro,. 2005;21(4):1186–200
187	Mooij SH, LandÈn O, van der Klis FRM, van der Sande MAB, de Melker HE, Coutinho RA, et al. No evidence for a protective effect of naturally induced HPV antibodies on subsequent anogenital HPV infection in HIV-negative and HIV-infected MSM. Journal of Infection. 2014;69(4):375–86
188	Moraska AR, Chamberlain AM, Shah ND, et al. Depression, Healthcare Utilization, and Death in Heart Failure. Circulation: Heart Failure. 2013;6(3):387–94
189	Morris AA, McAllister P, Grant A, et al. Relation of Living in a ìFood Desertî to Recurrent Hospitalizations in Patients With Heart Failure. The American Journal of Cardiology. 2019;123(2):291–6
190	Musoro JZ, Geskus RB, Zwinderman AH. A joint model for repeated events of different types and multiple longitudinal outcomes with application to a follow-up study of patients after kidney transplant. Biometrical Journal. 2014;57(2):185–200
191	N. Albers L, Liu Y, A. Swerlick R, J. Feldman R. Developing biomarkers for predicting clinical relapse in pemphigus patients treated with rituximab. American Academy of Dermatology. 2017
192	Nager A, Szulkin R, Johansson S-E, Johansson L-M, Sundquist K. High lifelong relapse rate of psychiatric disorders among women with postpartum psychosis. Nordic Journal of Psychiatry. 2012;67(1):53–8
193	Najafi-Vosough R, Reza Soltanian A, Fayyazi N. Influence Factors on Birth Spacing and Childbearing Rates using Survival Recurrent Events Model and Parity Progression Ratios. Journal of Research in Health Sciences. 2017;JRHS 2017; 17(3): e00384
194	Nathoo FS, Dean CB. Spatial Multistate Transitional Models for Longitudinal Event Data. Biometrics. 2007;64(1):271–9
195	Navarro A, Ancizu I. Analyzing the occurrence of falls and its risk factors: Some considerations. Preventive Medicine. 2009;48(3):298–302
196	Nessim SJ, Bargman JM, Austin PC, Nisenbaum R, Jassal SV. Predictors of Peritonitis in Patients on Peritoneal Dialysis: Results of a Large, Prospective Canadian Database. Clinical Journal of the American Society of Nephrology. 2009;4(7):1195–200
197	Neustifter B, Rathbun SL, Shiffman S. Mixed-Poisson point process with partially observed covariates: ecological momentary assessment of smoking. Journal of Applied Statistics. 2012;39(4):883–99
198	Ngure K, Heffron R, Mugo NR, et al. Contraceptive method and pregnancy incidence among women in HIV-1-serodiscordant partnerships. Aids. 2012;26(4):513–8
199	Nosyk B, Anglin MD, Brecht M-L, Lima VD, Hser Y-I. Characterizing Durations of Heroin Abstinence in the California Civil Addict Program: Results From a 33-Year Observational Cohort Study. American Journal of Epidemiology. 2013;177(7):675–82
200	Nosyk B, LourenÁo L, Min JE, Shopin D, Lima VD, Montaner JSG. Characterizing retention in HAART as a recurrent event process. Aids. 2015;29(13):1681–9
201	Nosyk B, MacNab YC, Sun H, Fischer B, Marsh DC, Schechter MT, et al. Proportional Hazards Frailty Models for Recurrent Methadone Maintenance Treatment. American Journal of Epidemiology. 2009;170(6):783–92
202	Nuti LA, Lawley M, Turkcan A, Tian Z, Zhang L, Chang K, et al. No-shows to primary care appointments: subsequent acute care utilization among diabetic patients. BMC Health Services Research. 2012;12(1)
203	Odio CD, Carroll M, Glass S, Bauman A, Taxman FS, Meyer JP. Evaluating concurrent validity of criminal justice and clinical assessments among women on probation. Health & Justice. 2018;6(1)
204	Oh WK, Proctor K, Nakabayashi M, Evan C, Tormey LK, Daskivich T, et al. The risk of renal impairment in hormone-refractory prostate cancer patients with bone metastases treated with zoledronic acid. Cancer. 2007;109(6):1090–6
205	Olotu A, Fegan G, Wambua J, et al. Four-Year Efficacy of RTS,S/AS01E and Its Interaction with Malaria Exposure. New England Journal of Medicine. 2013;368(12):1111–20
206	Oma E, Jensen KK, Bisgaard T, Jorgensen LN. Association of Primary Ventral Hernia and Pregnancy. Annals of Surgery. 2018;272(1):170–6
207	Osmani F, Hajizadeh E, Akbar Rasekhi A. Association between Multiple Recurrent Events with Multivariate Modeling: A Retrospective Cohort Study. Journal of Research in Health Sciences. 2018;18(4): e00433
208	Ouellet G, Huang DT, Moss AJ, et al. Effect of Cardiac Resynchronization Therapy on the Risk of First and Recurrent Ventricular Tachyarrhythmic Events in MADIT-CRT. Journal of the American College of Cardiology. 2012;60(18):1809–16
209	P.Piccini J, Mittal S, Snell J, et al. Impact of remote monitoring on clinical events and associated health care utilization: A nationwide assessment. Heart Rhythm Society. 2016
210	Pajewski NM, Williamson JD, Applegate WB, et al. Characterizing Frailty Status in the Systolic Blood Pressure Intervention Trial. The Journals of Gerontology Series A: Biological Sciences and Medical Sciences. 2016;71(5):649–55
211	Palmstierna T, Olsson D. Violence from young women involuntarily admitted for severe drug abuse. Acta Psychiatrica Scandinavica. 2007;115(1):66–72
212	Patel DK, Duncan MS, Shah AS, Lindman BR, Greevy RA, Savage PD, et al. Association of Cardiac Rehabilitation With Decreased Hospitalization and Mortality Risk After Cardiac Valve Surgery. JAMA Cardiology. 2019;4(12):1250
213	Pedersen J, Bjorner JB, Burr H, Christensen KB. Transitions between sickness absence, work, unemployment, and disability in Denmark 2004ñ2008. Scandinavian Journal of Work, Environment & Health. 2012;38(6):516–26
214	PÈnichoux J, Michiels S, BouchÈ O, et al. Taking into account successive treatment lines in the analysis of a colorectal cancer randomised trial. European Journal of Cancer. 2013;49(8):1882–8
215	PeÒa EA, Slate EH, Gonz·lez JR. Semiparametric inference for a general class of models for recurrent events. Journal of Statistical Planning and Inference. 2007;137(6):1727–47
216	Peprah S, Coleman JS, Rositch AF, Vanden Bussche CJ, Moore R, DíSouza G. Utilization of Pap testing among women living with HIV enrolled in primary care in Baltimore, Maryland: A 10-year longitudinal study, 2005ñ2014. Papillomavirus Research. 2018;6:52–7
217	Persons JE, Coryell WH, Solomon DA, Keller MB, Endicott J, Fiedorowicz JG. Mixed state and suicide: Is the effect of mixed state on suicidal behavior more than the sum of its parts? Bipolar Disorders. 2017;20(1):35–41
218	Pfennig A, Schlattmann P, Alda M, Grof P, Glenn T, M¸ller-Oerlinghausen B, et al. Influence of atypical features on the quality of prophylactic effectiveness of long-term lithium treatment in bipolar disorders. Bipolar Disorders. 2010;12(4):390–6
219	Pintye J, Baeten JM, Manhart LE, et al. Association between male circumcision and incidence of syphilis in men and women: a prospective study in HIV-1 serodiscordant heterosexual African couples. The Lancet Global Health. 2014;2(11):e664-e71
220	Pohl P, Nordin E, Lundquist A, Bergstrˆm U, Lundin-Olsson L. Community-dwelling older people with an injurious fall are likely to sustain new injurious falls within 5†years—a prospective long-term follow-up study. BMC Geriatrics. 2014;14(1)
221	Pontes MV, Ribeiro TCM, Ribeiro H, et al. Cowís milk-based beverage consumption in 1- to 4-year-olds and allergic manifestations: an RCT. Nutrition Journal. 2015;15(1)
222	Putter H, van der Hage J, de Bock GH, Elgalta R, van de Velde CJH. Estimation and Prediction in a Multi-State Model for Breast Cancer. Biometrical Journal. 2006;48(3):366–80
223	Rahmati M, Rahgozar M, Fadaei F, Bakhshi E, Cheraghi L. Identifying Some Risk Factors of Time to Relapses in Schizophrenic Patients using Bayesian Approach with Event-Dependent Frailty Model. Iran J Psychiatry. 2015;10(2):P123-7
224	Ramhormozi SM, Moghimbeigi A, Mahjub H, Soltanian AR. Birth Distance Influential Factors: A Multilevel Recurrent Events Approach. Journal of Research in Health Science. 2010;10(2):98–103
225	Rana AI, Liu T, Gillani FS, Reece R, Kojic EM, Zlotnick C, et al. Multiple gaps in care common among newly diagnosed HIV patients. AIDS Care. 2015;27(6):679–87
226	Rancoita PMV, Valberg M, Demicheli R, Biganzoli E, Di Serio C. Tumor dormancy and frailty models: A novel approach. Biometrics. 2016;73(1):260–70
227	Rassouli F, Baty F, Stolz D, Albrich W, Tamm M, Widmer S, et al. Longitudinal change of COPD assessment test (CAT) in a telehealthcare cohort is associated with exacerbation risk. International Journal of Chronic Obstructive Pulmonary Disease. 2017;Volume 12:3103–9
228	Rathbun SL, Shiffman S. Mixed effects models for recurrent events data with partially observed time-varying covariates: Ecological momentary assessment of smoking. Biometrics. 2015;72(1):46–55
229	Rice JD, Johnson BA, Strawderman RL. Modeling the rate of HIV testing from repeated binary data amidst potential never-testers. Biostatistics. 2018;20(2):183–98
230	Robertsson O, Ranstam J. No bias of ignored bilaterality when analysing the revision risk of knee prostheses: Analysis of a population based sample of 44,590 patients with 55,298 knee prostheses from the national Swedish Knee Arthroplasty Register. BMC Musculoskeletal Disorders. 2003;4(1)
231	Rogers JK, Jhund PS, Perez A-C, et al. Effect of Rosuvastatin on Repeat Heart Failure Hospitalizations. JACC: Heart Failure. 2014;2(3):289–97
232	Rondeau V, Gonzalez JR. frailtypack: A computer program for the analysis of correlated failure time data using penalized likelihood estimation. Computer Methods and Programs in Biomedicine. 2005;80(2):154–64
233	Ronen K, McCoy CO, Matsen FA, et al. HIV-1 Superinfection Occurs Less Frequently Than Initial Infection in a Cohort of High-Risk Kenyan Women. PLoS Pathogens. 2013;9(8):e1003593
234	Ruiz JG, Rodriguez-Suarez M, Tang F, Aparicio-Ugarriza R, Ferri-Guerra J, Mohammed NY, et al. Depression but not frailty contributed to a higher risk for all-cause hospitalizations in male older veterans. International Journal of Geriatric Psychiatry. 2019;35(1):37–44
235	Sadatsafavi M, Xie H, Etminan M, Johnson K, FitzGerald JM. The association between previous and future severe exacerbations of chronic obstructive pulmonary disease: Updating the literature using robust statistical methodology. Plos One. 2018;13(1):e0191243
236	Sagara I, Giorgi R, Doumbo OK, Piarroux R, Gaudart J. Modelling recurrent events: comparison of statistical models with continuous and discontinuous risk intervals on recurrent malaria episodes data. Malaria Journal. 2014;13(1)
237	Sapyta J, Goldston DB, Erkanli A, Daniel SS, Heilbron N, Mayfield A, et al. Evaluating the predictive validity of suicidal intent and medical lethality in youth. Journal of Consulting and Clinical Psychology. 2012;80(2):222–31
238	Sarkar S, Koehler J, Crossley GH, Tang WHW, Abraham WT, Warman EN, et al. Burden of atrial fibrillation and poor rate control detected by continuous monitoring and the risk for heart failure hospitalization. American Heart Journal. 2012;164(4):616–24
239	Saunders LL, Selassie AW, Hill EG, Nicholas JS, Horner MD, Corrigan JD, et al. A population-based study of repetitive traumatic brain injury among persons with traumatic brain injury. Brain Injury. 2009;23(11):866–72
240	Schaubel DE. Analysis of clustered recurrent event data with application to hospitalization rates among renal failure patients. Biostatistics. 2005;6(3):404–19
241	Seyoum D, Kifle YG, Rondeau V, Yewhalaw D, Duchateau L, Rosas-Aguirre A, et al. Identification of different malaria patterns due to Plasmodium falciparum and Plasmodium vivax in Ethiopian children: a prospective cohort study. Malaria Journal. 2016;15(1)
242	Shovlin CL, Jackson JE, Bamford KB, Jenkins IH, Benjamin AR, Ramadan H, et al. Primary determinants of ischaemic stroke/brain abscess risks are independent of severity of pulmonary arteriovenous malformations in hereditary haemorrhagic telangiectasia. Thorax. 2008;63(3):259–66
243	Sintek MA, Sparrow CT, Mikuls TR, Lindley KJ, Bach RG, Kurz HI, et al. Repeat revascularisation outcomes after percutaneous coronary intervention in patients with rheumatoid arthritis. Heart. 2015;102(5):363–9
244	Smedinga H, Steyerberg EW, Beukers W, van Klaveren D, Zwarthoff EC, Vergouwe Y. Prediction of Multiple Recurrent Events: A Comparison of Extended Cox Models in Bladder Cancer. American Journal of Epidemiology. 2017;186(5):612–23
245	Smolderen KG, Plomondon ME, Armstrong EJ, Hess E, Waldo S, Tsai TT, et al. Depression and long-term prognostic outcomes following peripheral endovascular interventions in the VA Healthcare System. Vascular Medicine. 2018;23(5):454–60
246	Snipelisky D, Stulak JM, Schettle SD, Sharma S, Kushwaha SS, Dunlay SM. Psychosocial characteristics and outcomes in patients with left ventricular assist device implanted as destination therapy. American Heart Journal. 2015;170(5):887–94
247	Sokoreli I, Pauws SC, Steyerberg EW, et al. Prognostic value of psychosocial factors for first and recurrent hospitalizations and mortality in heart failure patients: insights from the OPERA-HF study. European Journal of Heart Failure. 2018;20(4):689–96
248	Solomon SD, Adams D, Kristen A, et al. Effects of Patisiran, an RNA Interference Therapeutic, on Cardiac Parameters in Patients With Hereditary Transthyretin-Mediated Amyloidosis. Circulation. 2019;139(4):431–43
249	Stephenson J, Farndon L, Concannon M. Analysis of a trial assessing the long-term effectiveness of salicylic acid plasters compared with scalpel debridement in facilitating corn resolution in patients with multiple corns. The Journal of Dermatology. 2015;43(6):662–9
250	Sudenga SL, Shrestha S, Macaluso M, Partridge EE, Johanning GL, Piyathilake CJ. Functional variants in CYP1A1 and GSTM1 are associated with clearance of cervical HPV infection. Gynecologic Oncology. 2014;135(3):560–4
251	Sudenga SL, Wiener HW, King CC. Dense Genotyping of Immune-Related Loci Identifies Variants Associated with Clearance of HPV among HIV-Positive Women in the HIV Epidemiology Research Study (HERS). PLoS ONE. 2014;9(6):e99109
252	Sutradhar R, Lokku A, Barbera L. Cancer survivorship and opioid prescribing rates: A population-based matched cohort study among individuals with and without a history of cancer. Cancer. 2017;123(21):4286–93
253	Tahiri M, Sikder T, Maimon G, et al. The impact of postoperative complications on the recovery of elderly surgical patients. Surgical Endoscopy. 2015;30(5):1762–70
254	Tavakol N, Kheiri S, Sedehi M. Analysis of the Factors Affecting the Interval between Blood Donations Using Log-Normal Hazard Model with Gamma Correlated Frailty. Journal of Research in Health Sciences (JHRS). 2016;16(2):76–80
255	Tawiah R, McLachlan GJ, Ng SK. Mixture cure models with time-varying and multilevel frailties for recurrent event data. Statistical Methods in Medical Research. 2019;29(5):1368–85
256	Tawiah R, Yau KKW, McLachlan GJ, Chambers SK, Ng S-K. Multilevel model with random effects for clustered survival data with multiple failure outcomes. Statistics in Medicine. 2018;38(6):1036–55
257	Taylor DH, Fillenbaum GG, Ezell ME. The accuracy of medicare claims data in identifying Alzheimer's disease. Journal of Clinical Epidemiology. 2002;55(9):929–37
258	Teerlink JR, Qian M, Bello NA, et al. Aspirin Does Not Increase Heart Failure†Events in Heart Failure Patients. JACC: Heart Failure. 2017;5(8):603–10
259	Teichert M, van Noord C, Uitterlinden AG, Hofman A, Buhre PN, De Smet PAGM, et al. Proton pump inhibitors and the risk of overanticoagulation during acenocoumarol maintenance treatment. British Journal of Haematology. 2011;153(3):379–85
260	Teichert M, Visser LE, Uitterlinden AG, Hofman A, Buhre PJ, Straus S, et al. Selective serotonin re-uptake inhibiting antidepressants and the risk of overanticoagulation during acenocoumarol maintenance treatment. British Journal of Clinical Pharmacology. 2011;72(5):798–805
261	Thangaratinam S, Marlin N, Newton S, et al. AntiEpileptic drug Monitoring in PREgnancy (EMPiRE): a double-blind randomised trial on effectiveness and acceptability of monitoring strategies. Health Technology Assessment. 2018;22(23):1–152
262	Tor·-Rocamora I, Gimeno D, Delclos G, Benavides FG, Manzanera R, JardÌ J, et al. Heterogeneity and event dependence in the analysis of sickness absence. BMC Medical Research Methodology. 2013;13(1)
263	Tuomaala S, Eskelin S, Tarkkanen A, Kivela T. Population-Based Assessment of Clinical Characteristics Predicting Outcome of Conjunctival Melanoma in Whites. Investigative Ophthalmology & Visual Science. 2002;43(11)
264	Tyblova M, Kalincik T, Zikan V, Havrdova E. Impaired ambulation and steroid therapy impact negatively on bone health in multiple sclerosis. European Journal of Neurology. 2014;22(4):624–32
265	Ullah S, Gabbett TJ, Finch CF. Statistical modelling for recurrent events: an application to sports injuries. British Journal of Sports Medicine. 2012;48(17):1287–93
266	van Strien AM, Souverein PC, Keijsers CJPW, Heerdink ER, Derijks HJ, van Marum RJ. Association Between Urinary Tract Infections and Antipsychotic Drug Use in Older Adults. Journal of Clinical Psychopharmacology. 2018;38(4):296–301
267	Vanhoutte B. Age Takes Hold of Us by Surprise: Conceptualizing Vulnerabilities in Aging as the Timing of Adverse Events. The Journals of Gerontology: Series B. 2019;76(1):152–60
268	Vasudevan A, Choi JW, Feghali GA, et al. First and recurrent events after percutaneous coronary intervention: implications for survival analyses. Scandinavian Cardiovascular Journal. 2019;53(6):299–304
269	Vasudevan A, Choi JW, Feghali GA, et al. Event dependence in the analysis of cardiovascular readmissions postpercutaneous coronary intervention. Journal of Investigative Medicine. 2019;67(6):943–9
270	Vigan M, Stirnemann J, MentrÈ F. Evaluation of Estimation Methods and Power of Tests of Discrete Covariates in Repeated Time-to-Event Parametric Models: Application to Gaucher Patients Treated by Imiglucerase. The AAPS Journal. 2014;16(3):415–23
271	Visser M, Heijne JCM, Hogewoning AA, van Aar F. Frequency and determinants of consistent STI/HIV testing among men who have sex with men testing at STI outpatient clinics in the Netherlands: a longitudinal study. Sexually Transmitted Infections. 2017;93(6):396–403
272	Wall KM, Kilembe W, Vwalika B, et al. Sustained effect of couples' HIV counselling and testing on risk reduction among Zambian HIV serodiscordant couples. Sexually Transmitted Infections. 2017;93(4):259–66
273	Wand H, Ramjee G. Biological impact of recurrent sexually transmitted infections on HIV seroconversion among women in South Africa: results from frailty models. Journal of the International AIDS Society. 2015;18(1):19,866
274	Wang K, Yau KKW, Lee AH, McLachlan GJ. Multilevel survival modelling of recurrent urinary tract infections. Computer Methods and Programs in Biomedicine. 2007;87(3):225–9
275	Wang M, Szepietowska B, Polonsky B, McNitt S, Moss AJ, Zareba W, et al. Risk of Cardiac Events Associated With Antidepressant Therapy in Patients With Long QT Syndrome. The American Journal of Cardiology. 2018;121(2):182–7
276	Weaver A, Troum O, Hooper M, Koenig AS, Chaudhari S, Feng J, et al. Rheumatoid Arthritis Disease Activity and Disability Affect the Risk of Serious Infection Events in RADIUS 1. The Journal of Rheumatology. 2013;40(8):1275–81
277	Wen S, Huang X, Frankowski RF, Cormier JN, Pisters P. A Bayesian multivariate joint frailty model for disease recurrences and survival. Statistics in Medicine. 2016;35(26):4794–812
278	Westbury LD, Syddall HE, Simmonds SJ, Cooper C, Sayer AA. Identification of risk factors for hospital admission using multiple-failure survival models: a toolkit for researchers. BMC Medical Research Methodology. 2016;16(1)
279	Weycker D, Edelsberg J, Barron R, Atwood M, Oster G, Crittenden DB, et al. Predictors of near-term fracture in osteoporotic women aged = 65†years, based on data from the study of osteoporotic fractures. Osteoporosis International. 2017;28(9):2565–71
280	Weycker D, Li X, Wygant GD, et al. Effectiveness and Safety of Apixaban versus Warfarin as Outpatient Treatment of Venous Thromboembolism in U.S. Clinical Practice. Thrombosis and Haemostasis. 2018;118(11):1951–61
281	Whang W, Albert CM, Sears SF, Lampert R, Conti JB, Wang PJ, et al. Depression as a predictor for appropriate shocks among patients with implantable cardioverter-defibrillators. Journal of the American College of Cardiology. 2005;45(7):1090–5
282	Willemze RA, Bakker T, Pippias M, Ponsioen CY, de Jonge WJ. fl-Blocker use is associated with a higher relapse risk of inflammatory bowel disease. European Journal of Gastroenterology & Hepatology. 2018;30(2):161–6
283	Wu W-T, Tsai S–S, Liao H-Y, et al. Usefulness of overnight pulse oximeter as the sleep assessment tool to assess the 6-year risk of road traffic collision: evidence from the Taiwan Bus Driver Cohort Study. International Journal of Epidemiology. 2016:dyw141
284	Xia H, Ebben J, Ma JZ, Collins AJ. Hematocrit Levels and Hospitalization Risks in Hemodialysis Patients. Journal of the American Society of Nephrology. 1999;10(6):1309–16
285	Xu C, Chinchilli VM, Wang M. Joint modeling of recurrent events and a terminal event adjusted for zero inflation and a matched design. Statistics in Medicine. 2018;37(18):2771–86
286	Xu J, Lam KF, Chen F, Milligan P, Cheung YB. Semiparametric estimation of time-varying intervention effects using recurrent event data. Statistics in Medicine. 2017;36(17):2682–96
287	Xu L, Yu C, Jiang J, et al. Major adverse cardiac events in elderly patients with coronary artery disease undergoing noncardiac surgery: A multicenter prospective study in China. Archives of Gerontology and Geriatrics. 2015;61(3):503–9
288	Yan Y, Andriole GL, Humphrey PA, Kibel AS. Patterns of multiple recurrences of superficial (Ta/T1) transitional cell carcinoma of bladder and effects of clinicopathologic and biochemical factors. Cancer. 2002;95(6):1239–46
289	Ye Y, Kalbfleisch JD, Schaubel DE. Semiparametric Analysis of Correlated Recurrent and Terminal Events. Biometrics. 2007;63(1):78–87
290	Yonekura S, Terauchi F, Hoshi K, Yamaguchi T, Kawai S. Androgen Receptor Predicts First and Multiple Recurrences in Non-Muscle Invasive Urothelial Carcinoma of the Bladder. Pathology & Oncology Research. 2018;25(3):987–94
291	Young JT, Arnold-Reed D, Preen D, Bulsara M, Lennox N, Kinner SA. Early primary care physician contact and health service utilisation in a large sample of recently released ex-prisoners in Australia: prospective cohort study. BMJ Open. 2015;5(6):e008021-e
292	Young JT, Borschmann R, Preen DB, Spittal MJ, Brophy L, Wang EA, et al. Age-specific incidence of injury-related hospital contact after release from prison: a prospective data-linkage study. Injury Prevention. 2019;26(3):204–14
293	Yu B. A Frailty Mixture Cure Model with Application to Hospital Readmission Cata. Biometrical Journal. 2008;50(3):386–94
294	Yu H, Cheng Y-J, Wang C-Y. Semiparametric Regression Estimation for Recurrent Event Data with Errors in Covariates under Informative Censoring. The International Journal of Biostatistics. 2016;12(2)
295	Yu H, Cheng Y-J, Wang C-Y. Methods for multivariate recurrent event data with measurement error and informative censoring. Biometrics. 2018;74(3):966–76
296	Yu Z, Liu L, Bravata DM, Williams LS. Joint model of recurrent events and a terminal event with time-varying coefficients. Biometrical Journal. 2013;56(2):183–97
297	Yu Z, Liu L, Bravata DM, Williams LS, Tepper RS. A semiparametric recurrent events model with time-varying coefficients. Statistics in Medicine. 2012;32(6):1016–26
298	Zaleski M, Gogoj A, Walter V. Mitotic activity in noninvasive papillary urothelial carcinoma: its value in predicting tumor recurrence and comparison with the contemporary 2-tier grading system. Human Pathology. 2019;84:275–82
299	Zanolla L, Guarise P, Tomasi L. Association between Beta1-Adrenergic Receptor Polymorphism and Risk of ICD Shock in Heart Failure Patients. Wiley. 2016;PACE, Vol. 39
300	Zhang B, Lin H, Hunter DJ, Neogi T, Wise B, Choy E, et al. A Multistate Transition Model for Osteoarthritis Pain Change. Communications in Statistics—Theory and Methods. 2009;38(18):3297–306
301	Zhao X, Liu L, Liu Y, Xu W. Analysis of multivariate recurrent event data with time-dependent covariates and informative censoring. Biometrical Journal. 2012;54(5):585–99

## Appendix 3

Clinical area recurrent event methods applied in

**Table 13:** Frequency of clinical area recurrent event methods applied in

**Clinical area**	**Number (%) of included studies**^1,2^
Cardiology	62 (20.6%)
Oncology	45 (15.0%)
Human immunodeficiency virus (HIV)/Acquired immunodeficiency syndrome (AIDS)	30 (10.0%)
Mental health	24 (8.0%)
Neurology	21 (7.0%)
Kidney disease	13 (4.3%)
Respiratory	11 (3.7%)
Drugs & alcohol abuse	9 (3.0%)
Elderly people & accidents	9 (3.0%)
Infectious diseases	9 (3.0%)
Other illness	9 (3.0%)
Sexually transmitted infection (STI)/Sexually transmitted disease (STD)	9 (3.0%)
Paediatrics	8 (2.7%)
Haematology	5 (1.7%)
Hospital admissions	5 (1.7%)
Arthritis	4 (1.3%)
Diabetes	4 (1.3%)
Maternal health	4 (1.3%)
Chronic injuries	3 (1.0%)
Surgeries	3 (1.0%)
Bacterial infections	2 (0.7%)
Gastroenterology	2 (0.7%)
Optometry	2 (0.7%)
Osteoarthropathy	2 (0.7%)
Autoimmune Disease	1 (0.3%)
Gynecology	1 (0.3%)
Inflammatory bowel disease	1 (0.3%)
Ophthalmology	1 (0.3%)
Podiatry	1 (0.3%)

^1^Some studies applied recurrent event analysis models to more than one clinical area

^2^Results are sorted in descending frequency

## Abbreviations

AIC: Akaike information criterion
AIDS: Acquired immunodeficiency syndrome
AG: Andersen-Gill
BIC: Bayesian information criterion
DIC: Deviance information criterion
EPV: Events per variable
HIV: Human immunodeficiency virus
IQR: Interquartile-range
LN: Lawless and Nadeau marginal model
LOCF: Last Observation Carried Forward
LSC: Liang, Self and Chang
LTF: Lost to follow-up
LWA: Lee, Wei and Amato
LWYY: Lin, Wei, Ying and Yang
MAPE: Mean absolute percentage error
MEDLINE: Medical Literature Analysis and Retrieval System Online
MSE: Mean square error
MSM: Multi-state model
PRISMA: Preferred-Reporting Items for Systematic Reviews and Meta-Analyses
PROBAST: Prediction model Risk Of Bias ASsessment Tool
PROSPERO: International Prospective Register of Systematic Reviews
PWP -GT: Prentice, Williams and Peterson–Gap Time
PWP -TT: Prentice, Williams and Peterson–Total Time
RCT: Randomised controlled trial
RMSE: Root mean square error
RMPSE: Root mean square percentage error
SGF: Shared gamma frailty
STD: Sexually transmitted disease
STI: Sexually transmitted infection
TRIPOD: Transparent reporting of a multivariable prediction model for individual prognosis or diagnosis
WHO: World Health Organization
WLW: Wei, Lei and Weissfeld
